# A Case of Periodontal Necrosis following Embolization of Maxillary Artery for Epistaxis

**DOI:** 10.1155/2016/6467974

**Published:** 2016-11-21

**Authors:** Kohei Nishimoto, Ryosei Minoda, Ryoji Yoshida, Toshinori Hirai, Eiji Yumoto

**Affiliations:** ^1^Department of Otolaryngology Head and Neck Surgery, Graduate School of Medicine, Kumamoto University, Kumamoto, Japan; ^2^Department of Oral and Maxillofacial Surgery, Graduate School of Medicine, Kumamoto University, Kumamoto, Japan; ^3^Department of Diagnostic Radiology, Graduate School of Medicine, Kumamoto University, Kumamoto, Japan

## Abstract

Embolization of the maxillary artery (MA) is a common treatment modality for refractory epistaxis. Tissue necrosis after embolization of the MA is a rare complication. Here, we reported the first case of the development of necrosis of soft tissue and alveolar bone in the periodontium after embolization. A 48-year-old man with poor oral hygiene and a heavy smoking habit was referred to our clinic due to intractable epistaxis. After treatment with anterior-posterior nasal packing (AP nasal packing), the epistaxis relapsed. Therefore, he underwent embolization of the MA. Although he did not experience epistaxis after embolization, periodontal necrosis developed gradually. The wound healed with necrotomy, administration of antibiotics and prostaglandin, and hyperbaric oxygen therapy. We speculated that the periodontal necrosis was provoked by reduction of blood supply due to embolization and AP nasal packing based on this preexisting morbid state in the periodontium. Poor condition of the oral cavity and smoking may increase the risk of periodontal necrosis after embolization.

## 1. Introduction

Epistaxis is a common medical problem, occurring in approximately 60% of the population at some time in their life [1–3]. Only 6% of patients with epistaxis require professional medical attention [4]. However, posterior or superior bleeding can often result in intractable epistaxis. Since Sokoloff et al. first reported selective angiography with embolization of the maxillary artery (MA) for treatment of intractable epistaxis in 1974 [5], this technique has gained increased acceptance as a safe and effective treatment for posterior nasal bleeding, with reported success rates of 77.3–94.6%, taking early rebleeding into account [1]. The incidence rate of major complications was reported to be 0–2% [1–3, 6–10]. The reported complications include necrosis of facial skin, nasal alar cartilage, and hard palate mucosa, ischemic sialadenitis of the parotid and submandibular glands, facial scarring following ischemia, temporary hemiparesis, monocular visual field loss and blindness, peripheral facial nerve paralysis, and cerebral infarction. However, necrosis in the alveolar bone and the surrounding soft tissue in the periodontium after embolization of the MA have not been reported. Here, we report on the first case of necrosis developing in the soft tissue and alveolar bone in the periodontium after embolization.

## 2. Case Report

**Figure 1:**
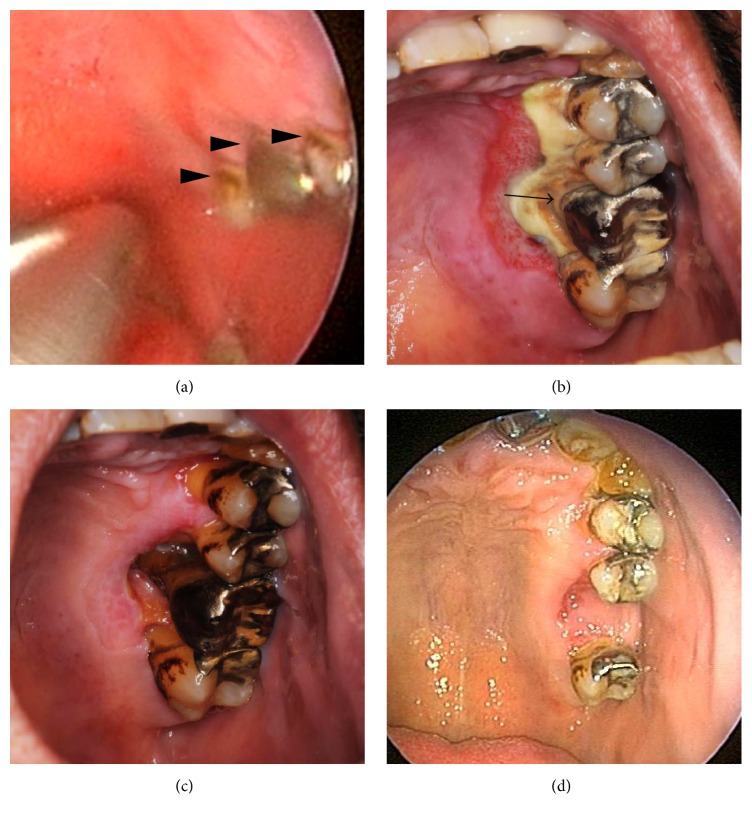
Temporal changes in the gingiva around the left upper teeth. (a) At the initial visit, plaque and calculus were observed on the tooth surface (arrowhead), and pockets had developed especially around his maxillary left first molar, which had been capped in silver. (b) Nine days after embolization, necrotizing ulcer formation was observed on the gingiva on the palatal side of the maxillary left premolar and molar teeth. The alveolar bone was exposed due to loss of gingiva (arrow). (c) At 2 weeks after treatment, resorption of the alveolar bone had occurred, and the palatal side of the first molar was fully exposed. Granulation tissue proliferated around the edge of the defect. (d) At 2 months after treatment, the wound was replaced by granulation tissue and epithelialized almost completely, with the exception of the deep socket on the palatal side.

A 48-year-old man was referred to our clinic due to intractable epistaxis on the left side. Although the patient had no remarkable medical history, he had been smoking 20 cigarettes per day for 30 years and had received no medical checkups or dental care for many years. On arrival, the source of bleeding was not visible due to continuous bleeding, and his blood pressure was significantly high (194/125 mmHg). Upon intraoral examination, marked accumulation of plaque and calculus was observed at the gingival margin of his maxillary left premolar and molar teeth (Figure 1(a)). Blood test revealed no significant abnormalities, including diabetes. The bleeding was stopped by nasal packing and nasopharyngeal balloon (hereafter referred to as anterior-posterior nasal packing: AP nasal packing). After hospitalization, a cardiologist started treatment for hypertension and his systolic blood pressure (SBP) decreased to 140–160 mmHg with 40 mg of nifedipine and 1.25 mg of bisoprolol fumarate, and then the pack was removed 5 days after packing. However, 2 days later, epistaxis relapsed, and he was referred to the interventional radiology division for angiography on the same day.

**Figure 2:**
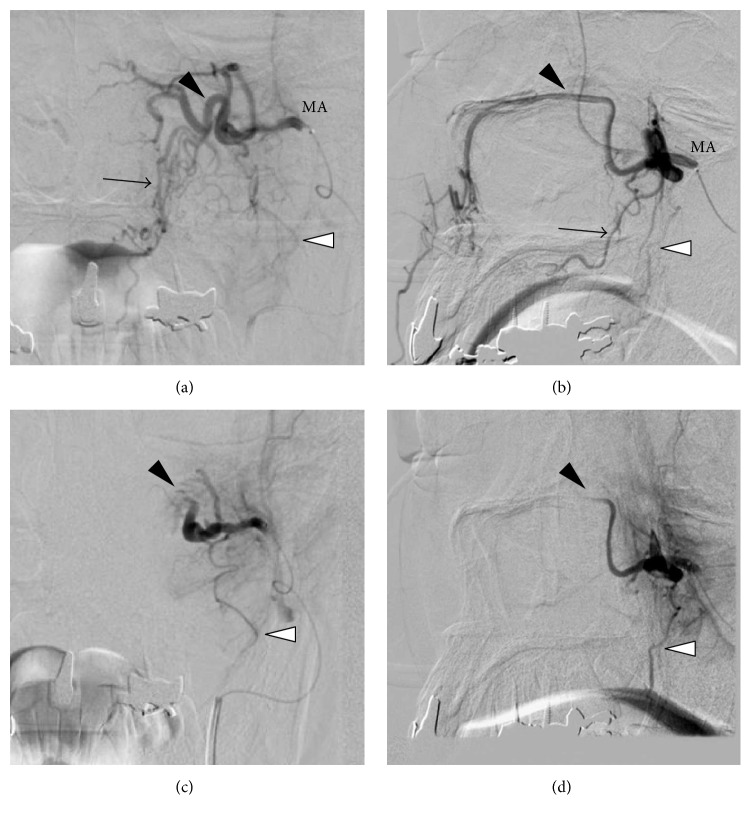
Digital subtraction angiography of the left maxillary artery (MA) in AP view (a, c) and lateral view (b, d). (a, b) Before embolization, well-developed sphenopalatine artery (SPA, black arrowhead) and descending palatine artery (DPA, arrow) were detected. (c, d) After embolization, the SPA and DPA disappeared, while the posterior superior alveolar artery (white arrowhead) was preserved.

Angiography of the external carotid artery revealed a well-developed left sphenopalatine artery (SPA) and retained contrast medium in the nose, but there was no extravasation. The left MA was selectively embolized using porous cellulose beads (PCBs; Asahi-Kasei, Tokyo, Japan) 230 and 400 *μ*m in diameter. After embolization, the SPA and descending palatine artery (DPA) disappeared (Figure 2). There was no bleeding after the procedure, although the patient reported slight pain in the left upper teeth, which increased gradually. On the day after removal of the AP nasal packing, the SBP was well controlled to around 120 mmHg by adding 80 mg of valsartan. He was discharged from the hospital 5 days after embolization because of no further epistaxis.

**Figure 3:**
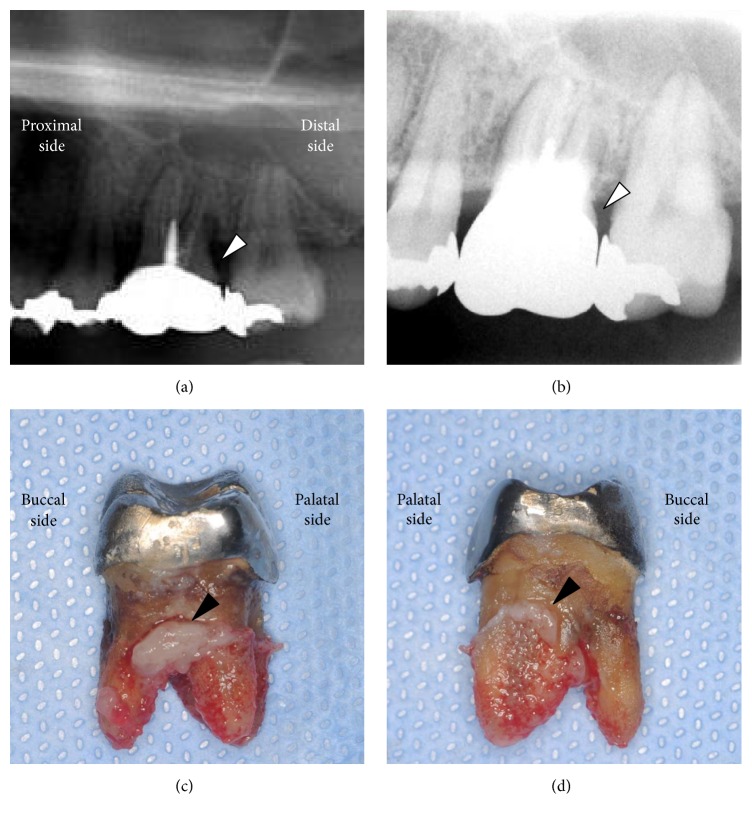
(a, b) Panoramic and dental radiographic images after embolization. The maxillary left first molar with crown and root filling material had a carious cavity on the distal side (white arrowhead). There was no defect in the alveolar bone. (c) The proximal and (d) distal sides of the extracted tooth. Cavities, remarkable calculus accumulation on the root surface, and infectious granuloma (arrowhead) were found.

Nine days after embolization, he visited the dental clinic in our hospital because of increasing tooth pain. Marked gingival necrosis was observed on the palatal side of the left maxillary premolar and molar, and the alveolar bone was exposed due to the loss of gingiva (Figure 1(b)). The buccal side was intact and there was no tooth mobility. Panoramic and dental radiographic images detected a carious cavity, but no defects in the alveolar bone (Figures 3(a) and 3(b)). He was hospitalized again and treated with necrotomy of the gingiva under local anesthesia preserving the alveolar bone, intravenous injection of ceftriaxone sodium (2 g/day), clindamycin (1200 mg/day), and prostaglandin E1 (120 *µ*g/day) and hyperbaric oxygen therapy for 2 weeks. The severe pain was controlled well by oral tramadol hydrochloride/acetaminophen. While granulation occurred gradually around the wound, resorption of the alveolar bone occurred, and the palatal side of the first molar was fully exposed (Figure 1(c)). At 3 weeks after admission, the maxillary left first molar showed mobility, and the tooth was extracted. The extracted tooth had cavities and marked calculus accumulation on the root surface (Figures 3(c) and 3(d)), indicating periodontitis that may have developed for several years. Two months after embolization, the wound was covered by granulation tissue and had epithelialized almost completely, with the exception of the deep socket on the palatal side (Figure 1(d)).

## 3. Discussion

The MA, which branches from the external carotid artery, is divided into three parts; the portion in the pterygopalatine fossa is called the “third portion” or pterygopalatine portion [11]. In this section, the MA enters through the pterygomaxillary fissure and branches into five arteries; in order before entering the sphenopalatine foramen, these are the posterior superior alveolar artery (PSAA) and the infraorbital artery branch off first and then the DPA, the artery of the pterygoid canal, and the SPA rise [11, 12]. The DPA branches into the greater and lesser palatine arteries and receives branches from the ascending palatal artery originating from the facial artery and the ascending pharyngeal artery [13, 14] and the greater palatine artery anastomoses with SPA at the nasal septum [15]. The palatal side of the upper periodontium is supplied by branches from the SPA (incisor and canine teeth) and greater palatine (premolar and molar teeth) arteries, while the premolar and molar teeth are supplied by the PSAA [15]. In our case, the third portion of MA was embolized, and consequently the SPA and the DPA disappeared on angiography while the PSAA remained. The feeding vessel of the molar tooth had been extirpated in previous root canal therapy and the simple loss of blood supply did not cause tooth loss. Embolization of the SPA and the DPA, which are feeding arteries of the palatal side of the upper periodontium, should cause loss of blood supply and/or tissue necrosis in the palatal side of the upper periodontium. Nevertheless, Pearson et al. reported that epistaxis patients did not show any tissue necrosis after ligation of the DPA and the proximal portion of the MA [16], and there have been no previous reports of periodontal necrosis after simple ligation or embolization of the MA and its branches. The blood supply in the palatal side of the periodontium should be maintained through the collateral blood supply from the ascending palatal artery and the ascending pharyngeal artery and through anastomosis between the greater palatine artery and the SPA, as mentioned above.

Guss et al. suggested that AP nasal packing likely exerts pressure on the soft palate and may cause reduction of blood flow to the greater palatine artery by compression of the ascending palatine artery and the ascending pharyngeal artery at the soft palate level [6]. This compression probably reduces the blood supply to the palatal side of the periodontium from the greater palatine artery and the SPA due to reduction of collateral blood supply from the ascending palatine artery and the ascending pharyngeal artery. The effect of reduction of blood supply to the palatal side of the periodontium by AP nasal packing may be more significant in cases where the proximal portions of the DPA and the SPA have been embolized or ligated. Indeed, Guss et al. reported one case in which intravascular embolization of the MA was performed along with AP nasal packing for 2 days [6], and the patient subsequently developed necrosis of the hard palate, which is also fed by the greater palatine artery [15]. Simultaneous embolization and AP nasal packing may also increase the risk of tissue necrosis in the periodontium, but not in the molar teeth, via a similar mechanism. Because of the patient's significantly high blood pressure, we maintained the AP packing for 5 days to decrease the risk of rebleeding. If we removed the AP packing before his blood pressure was normalized with medications, the rebleeding risk could be problematic. Changing the AP packing regimen might have resulted with a reduced risk of tissue necrosis in this case.

Polyvinyl alcohol (PVA), which is a nonabsorbable agent, is the most commonly used material for intravascular embolization [17]. PVA particles have a high friction coefficient due to the irregular surface, which permits the particles to rest against the wall without completely occluding the vessel, and sometimes they agglomerate in the delivery system itself [17]. This characteristic of PVA may cause incomplete filling of the vessel and may increase the probability of recanalization [17, 18]. In contrast, PCBs, which were used in our patient, are also nonabsorbable and exceptionally uniform in size. The nature of PCBs contributes to their smooth intravascular injection and complete embolization. Therefore, PCBs have longer occlusion ability and a low recanalization rate after embolization compared with PVA [17, 18]. Although the low recanalization rate of PCBs may decrease the possibility of relapse of epistaxis, the longer complete embolization by PCBs may increase the risk of tissue necrosis. Although we could not determine the precise locations of embolization in our patient, the distal portions of the SPA and the DPA appeared to be patent, and their blood flow was maintained from the collateral blood flow because the palatal side of the upper periodontium around the incisor and canine teeth, which are fed by branches of the SPA, were unaffected even after embolization. Thus, the use of PCBs was unlikely to be related to the local tissue necrosis in this case.

On the patient's first visit to our clinic, we found marked accumulation of plaque and calculus at the gingival margin of the patient's maxillary left premolar and molar teeth. These findings suggest that he had poor oral hygiene and had periodontitis for many years. Although he underwent dental treatment for these conditions after embolization, his periodontitis and tissue necrosis in the periodontium deteriorated, and he finally lost a tooth. Additionally, our patient had been smoking 20 cigarettes per day for 30 years. Smoking is known to reduce gingival blood flow and is a well-known aggravating factor for the development of periodontitis [19, 20]. His preexisting periodontitis and smoking habit would be major exacerbating factors for the progression of tissue necrosis in the periodontium and loss of the molar tooth. This preexisting morbid state in the periodontium of the premolar and molar teeth likely deeply affected the onset of tissue necrosis in our patient.

## 4. Conclusions

A side effect to embolization is necrosis of otherwise healthy tissue. This risk is increased if the patient has other vascular risks, as smoking, diabetes, or as in this case poor dental hygiene. We presented an epistaxis patient that developed tissue necrosis in the periodontium and loss of a molar tooth after intravascular embolization of the SPA and the DPA. Poor oral hygiene and smoking may increase the risk of periodontal necrosis after embolization. Furthermore, the period of AP nasal packing after embolization should be minimized to avoid reduction of blood supply in other areas.
